# Understanding the Role of the WRKY Gene Family under Stress Conditions in Pigeonpea (*Cajanus Cajan* L.)

**DOI:** 10.3390/plants8070214

**Published:** 2019-07-10

**Authors:** Akshay Singh, Pankaj Kumar Singh, Ajay Kumar Sharma, Nagendra Kumar Singh, Humira Sonah, Rupesh Deshmukh, Tilak Raj Sharma

**Affiliations:** 1 National Agri-Food Biotechnology Institute, Mohali, Punjab 140306 India; 2 Dr. A. P. J. Abdul Kalam Technical University, Lucknow, Uttar Pradesh 226031, India; 3 Meerut Institute of Engineering and Technology, Meerut, Uttar Pradesh 250005, India; 4 National Institute for Plant Biotechnology, New Delhi 110012, India

**Keywords:** transcription factors, WRKY domain, gene expressing profiling, stress tolerance, drought, high salinity

## Abstract

Pigeonpea (*Cajanus cajan* L.), a protein-rich legume, is a major food component of the daily diet for residents in semi-arid tropical regions of the word. Pigeonpea is also known for its high level of tolerance against biotic and abiotic stresses. In this regard, understanding the genes involved in stress tolerance has great importance. In the present study, identification, and characterization of WRKY, a large transcription factor gene family involved in numerous biological processes like seed germination, metabolism, plant growth, biotic and abiotic stress responses was performed in pigeonpea. A total of 94 *WRKY* genes identified in the pigeonpea genome were extensively characterized for gene-structures, localizations, phylogenetic distribution, conserved motif organizations, and functional annotation. Phylogenetic analysis revealed three major groups (I, II, and III) of pigeonpea *WRKY* genes. Subsequently, expression profiling of 94 *CcWRKY* genes across different tissues like root, nodule, stem, petiole, petal, sepal, shoot apical meristem (SAM), mature pod, and mature seed retrieved from the available RNAseq data identified tissue-specific *WRKY* genes with preferential expression in the vegetative and reproductive stages. Gene co-expression networks identified four *WRKY* genes at the center of maximum interaction which may play a key role in the entire *WRKY* regulations. Furthermore, quantitative real-time polymerase chain reaction (qRT-PCR) expression analysis of *WRKY* genes in root and leaf tissue samples from plants under drought and salinity stress identified differentially expressed *WRKY* genes. The study will be helpful to understand the evolution, regulation, and distribution of the *WRKY* gene family, and additional exploration for the development of stress tolerance cultivars in pigeonpea and other legumes crops.

## 1. Introduction

Pigeonpea (*Cajanus cajan* L.), a leguminous crop, belongs to tribe *Phaseoleae* of the family *Fabaceae*. Other members of the tribe *Phaseoleae* include common bean (*Phaseolus vulgaris*), soybean (*Glycine max*), cowpea (*Vigna ungiculata*), and mung bean (*Vigna radiata*) [1,2]. The term pigeonpea was originated in Barbados, where *Cajanus* seeds were used as pigeon-feed [3]. As per the Agricultural and Processed Food Products Export Development Authority (APEDA), 180,193.86 metric ton of pulses were exported to different countries of the world by India, worth 228.32 million US$ for the year 2017–18 [1,4]. Pigeonpea, an essential legume crop for the Indian subcontinent, is predominantly cultivated in tropical and subtropical regions worldwide. More than 85% of world pigeonpea production and usage takes place in India. Pigeonpea constitutes a principle component of Indian food and provides a rich source of proteins, vitamins, and minerals [5,6]. Pigeonpea, a drought-tolerant legume crop, is grown in locations with less than 300 mm yearly rainfall due to its extensive tap roots. Pigeonpea can grow in a wide range of temperature regimes from about 18 to over 30 °C [7]. Besides having a high level of stress tolerance, considerable yield losses occurred in pigeonpea due to the biotic and abiotic stress. The losses are mostly because of rainfed cultivation of pigeonpea where longer spells of dry seasons are frequent. 

Transcription factors (TFs) regulate the downstream expression of genes at the transcriptional level by binding to specific DNA sequences, thereby regulating and controlling various biological processes [8]. The WRKY family is known as the largest gene family across the higher plant species [9]. The WRKY family is known to have a significant role in stress tolerance mechanism in plants [10]. Numerous studies have shown a regulatory role of WRKY gene family members in biotic as well as abiotic stress tolerance in crop plants [11,12,13,14]. The family is named WRKY due to the occurrence of the domain containing well-conserved WRKYGQK domain at their N-terminus and a zinc figure structure (Cx_4–5_Cx_22–23_HxH or Cx_7_Cx_23_HxC) at their C-terminal. The length of the WRKY domain is approximately 60 amino acids long [15]. The WRKY protein domain consists of 4-stranded β-sheet and a zinc finger pocket that is responsible for the recognition with W-box sequence, (C/T)TGAC(T/C) [16]. On the basis of WRKY domain numbers and zinc-finger signature present, the WRKY proteins primarily classify into I, II and III groups. Members of group I contain two WRKY domains as well as a C_2_H_2_ zinc-finger pattern. Group II consists of a WRKY-domain along with a zinc-finger (C_2_H_2_) sequence. Group-II is further categorized into (IIa–IIe) subgroups. Additionally, the WRKY domain also presents in group III but contained a C_2_HC type zinc-finger sequence [17]. 

The first *WRKY* gene was cloned and characterized in 1994 from sweet potato [18]. Since then, cloning and characterization of several *WRKYs* have been performed in rice [19], wheat [20], cotton [21], soybean [22], tomato [23], banana [24] and the biofuel crop *Jatropha curcas* [25]. At the genome level, WRKY TFs family have also been analyzed in various crop species such as rice [26], common-bean [27], soybean [12], and chickpea [28].

The WRKY TFs plays a vital regulatory role in various developmental and physiological processes, such as seed germination [29], plant growth [30], secondary metabolism [31], trichome and seed development [32,33], panicle development [34], hormone signalling [35], leaf senescence [36], and bud and floral differentiation [37]. The WRKY TFs are also involved in a range of abiotic stress responses like cold [38], heat [39], drought as well as salinity [40,41], and biotic stresses for example fungi [42], bacteria [43], nematodes [44], viral pathogens [45], wounding [46], and aphid resistance [47]. 

Recent advances in next-generation sequencing (NGS) has provided numerous opportunities for plant genomics which includes transcriptome profiling, marker development, mapping loci diagnostic tools, evolutionary studies and most importantly whole genome sequencing [48,49,50,51]. Exploring the NGS tools, whole genome sequencing of pigeonpea cultivar Asha has been performed [5]. The publicly available well annotated genomic data provides a great opportunity to perform genome-wide studies which will help to understand the evolution of a particular gene family in pigeonpea lineage [52,53]. Similarly, RNAseq transcriptomics efforts has been performed in pigeonpea which serve as basis to study gene expression dynamics under different conditions and tissues [54,55].

In this study, 94 *WRKY* genes were identified in the pigeonpea genome and characterized for expression, conserved motifs, gene regulatory elements, gene ontology (GO) enrichment, and phylogenetic distribution. A relative expression study of identified *WRKY* genes in leaf and root tissues of two pigeonpea varieties (Asha-ICPL87119 and *Rynchosia minima*) was performed. Similarly, real-time polymerase chain reaction (PCR)-based expression profiling of selected *WRKY* genes under drought and salt stress treatments was performed to identify candidate genes having a role in stress tolerance. 

## 2. Results

### 2.1. Identification of WRKY Gene Family in Pigeonpea

Initially, a total of 97 *WRKY* gene family members were identified in pigeonpea genome with BLAST search performed using query sequences of known *WRKY* genes from ten different species. Out of 97 genes, 94 showed the occurrence of a well-conserved WRKY domain confirmed by NCBI-Conserved Domain Database searches (Figure S1). The WRKY proteins with no conserved WRKY domain were excluded from our analysis. Finally, 94 WRKY proteins which consist of a conserved WRKY domain were named as CcWRKY1 to CcWRKY94, as per their positions on *C. cajan* chromosome numbers 1–11 and scaffolds (Table S1). The length of CcWRKY proteins ranged between 84 to 678 amino acids (AA) with an average of 346 AA. From the 94 CcWRKY proteins, 83 have a highly conserved heptapeptide domain ‘WRKYGQK’, whereas ‘WRKYGKK’, ‘WRKYGEK’, ‘WRKYGRK’ or ‘WKKFEDK’ domains are present in the rest of the CcWRKY proteins (Figure S2). A total of 16 genes having two WRKY domains with (C2H2)-type zinc finger pattern classified as group-I. While the remaining 63 members contained a C2H2-type and 15 members contained a C2HC-type zinc finger, which were ordered as groups II and III respectively. The molecular mass of the predicted CcWRKY proteins ranged from 9884.1 to 73,688.8 kDa and also showed a broad range of isoelectric points ranged from basic (4.74 pKa) to acidic (10.09 pKa). The aliphatic index indicates the thermostability of proteins, which ranges from 36.6 to 81.9. The CcWRKY92 was found to be the most thermostable among the predicted WRKY proteins (Table S1).

### 2.2. Phylogenetic Distribution of CcWRKY Proteins

**Figure 1:**
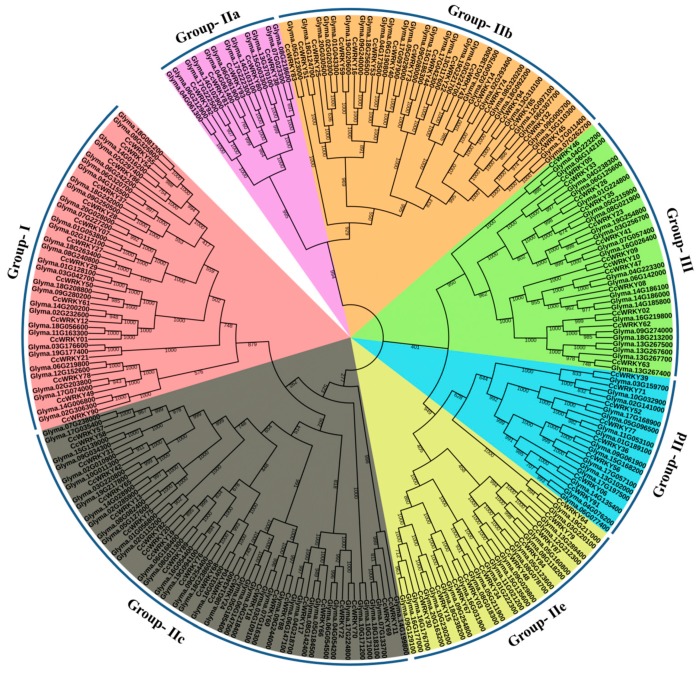
Unrooted phylogenetic tree of 267 WRKY proteins including 94 WRKY proteins from pigeonpea and 173 from soybean. Full-length amino acid sequences of WRKY from pigeonpea and soybean were aligned with Clustal X, and the phylogenetic tree was generated by the neighbor-joining method with 1000 bootstrap replicates using MEGA 7.0 software tool.

Based on the number of domains resembles within the combined phylogenetic tree of WRKY proteins from *Glycine max* and pigeonpea, the CcWRKY proteins were categorized into I, II and III groups (Figure 1). Group I has an independent clade, whereas groups II, III were shown to be quite similar to each other. Group II is further divided into five subgroups as previously observed in *Arabidopsis thaliana* [56]. Similarly, phylogenetic tree of 267 WRKY protein combined from *Glycine max*, *Phaseolus vulgaris* and *Arabidopsis thaliana* was constructed to confirm the groupings of WRKY members (Figure S3). Based on the tree of 267 WRKY from the four species, Group I and subgroup IIc of group II was found to be close, similarly, group IIa and IIb, and group IId and IIe found to be phylogenetically closer. 

**Figure 2:**
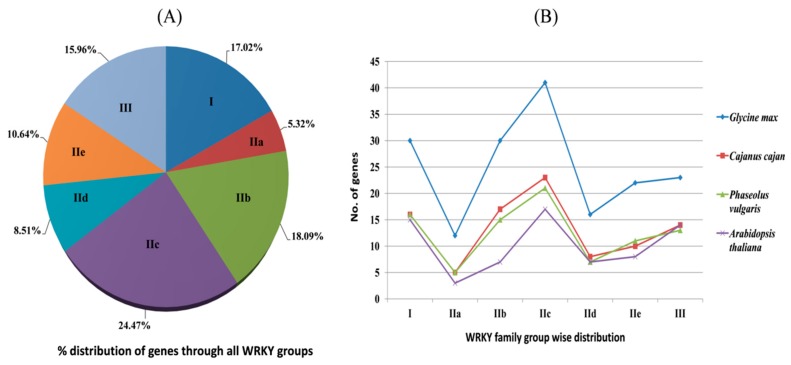
Distribution of WRKY group members. (**A**) Percentage of genes in different WRKY groups of pigeonpea genome, and (**B**) Comparison of *WRKY* genes distributed in different groups identified in pigeonpea and across different crop species including *Glycine max, Cajanus cajan, Phaseolus vulgaris*, and *Arabidopsis thaliana.*

The pigeonpea group I, subgroups IIa, IIb, IIc, IId, IIe and group III contained 17.0%, 5.3%, 18.1%, 24.4%, 8.5%, 10.6% and 15.9% of the total CcWRKY members, respectively (Figure 2A). Interestingly, CcWRKY11was found to be unique in pigeonpea which has a domain with sequence WKKFEDK present in the subgroup IIc, and very close to group I. Among the group II CcWRKY members, IIc has a relatively higher number of genes and IIa has fewer genes than the other sub-groups carried (Figure 2B).

### 2.3. Exon-Intron Organization and Motif Composition of WRKY Proteins

**Figure 3:**
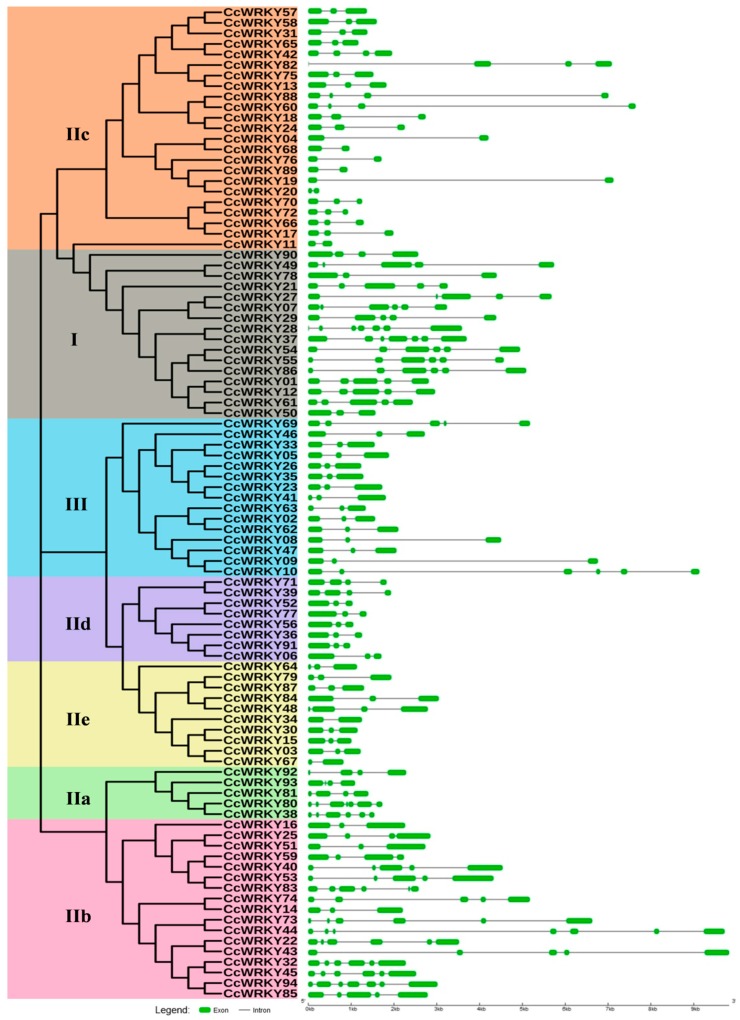
The intron-exon structure organization of 94 *WRKY* genes identified in pigeonpea genome. Exons and introns are indicated by solid green color boxes and solid black lines, respectively. The phylogenetic tree was constructed using the Neighbor-Joining method with 1000 bootstrap replicates.

The distribution of exon-intron was analyzed to gain more insight into the structural features of *CcWRKY* genes. The number of exon and intron varies from one to seven among *CcWRKY* gene categories (Figure 3). Among the 94 *CcWRKY* genes, 43 have three exons, 13 have four or five exons, followed by 11 *CcWRKYs* that had six exons, and five had seven exons. There were nine *CcWRKYs* containing only two exons, mainly belonging to the groups IIc and IIe. The *CcWRKY* genes family members from the same WRKY group commonly shared a similar gene structure in terms of intron/exon organization. For example, members of group III mostly had three exons, only *CcWRKY69* and *CcWRKY10* had five and six exons, respectively. In group II, exons ranged from two to six, except three genes that have seven exons (*CcWRKY44*, *CcWRKY80*, and *CcWRKY94*). However, the exon numbers in group I varied from two to seven.

**Figure 4:**
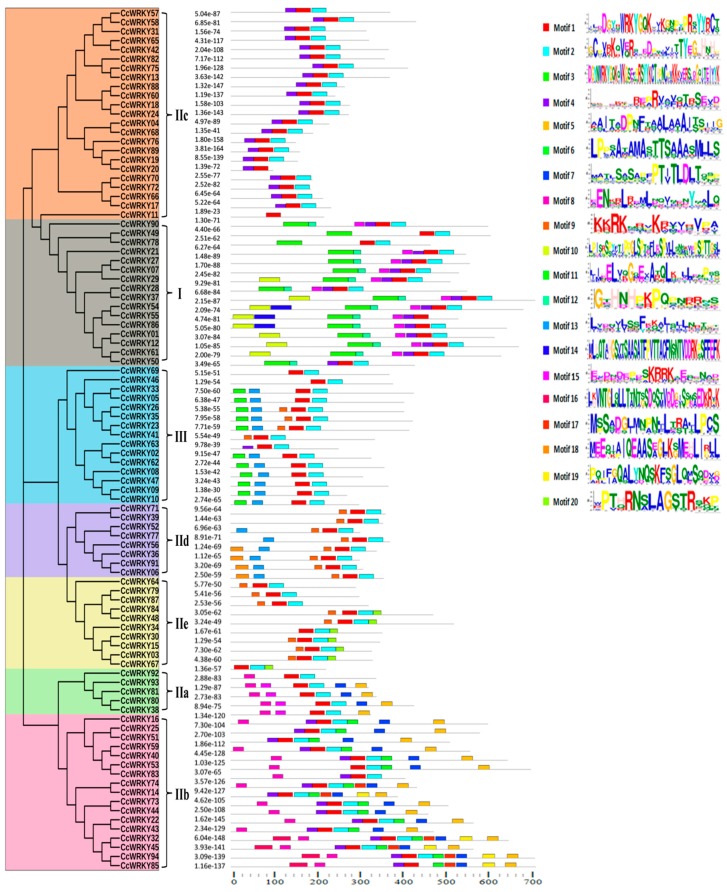
Schematic distribution of 20 conserved motifs predicted in 94 pigeonpea WRKY proteins. By using the MEME software tool (http://meme-suite.org), motif analysis of 94 WRKY protein identified in pigeonpea. The phylogenetic tree was constructed using the neighbor-joining method with 1000 bootstrap replicates.

For a better understanding, the diversity of CcWRKY protein motifs, the conserved motif analysis of CcWRKY protein sequences were investigated by using the online server MEME (http://meme.nbcr.net/meme/cgi-bin/meme.cgi) (Figure 4). From the total 20 predicted motifs, motif 1, as well as motif 3, consists of a WRKYGQK heptapeptide domain which is the unique feature of the WRKY TFs family. All the CcWRKY proteins consist of either motif 1 or motif 3, or both. The CcWRKY proteins that contained a similar type of motifs were clustered into a similar group in the phylogenetic tree. 

### 2.4. Stress-related Regulatory Elements in the Upstream Promoter Region and Functional Annotation of *WRKY* Genes

**Figure 5:**
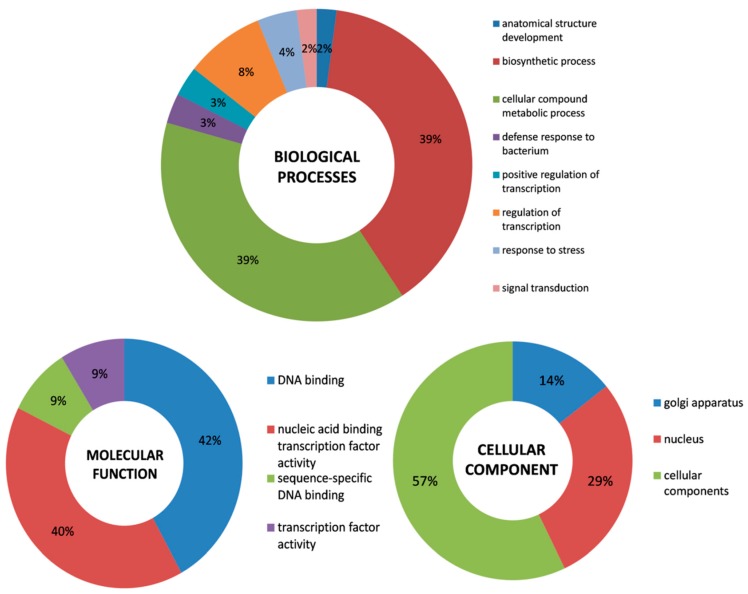
Functional annotation of the 94 WRKY proteins in pigeonpea. Distribution of functionally annotated genes in three main categories of GO classification viz. Biological processes, Molecular Function, and Cellular components.

Cis-regulatory elements are the binding sites of transcription factors responsible for transcriptional regulation of the gene. Usually, the regulatory region restricted to 5’ upstream (promoter) sequence of the gene [57]. Thus, the 2K upstream regulatory (proximal promoter) region from all the 94 *CcWRKY* genes was extracted and used to survey stress-responsive regulatory elements by using the online software PlantCARE. Only 53 bases from the proximal promoter region of *CcWRKY63* was extracted because it is present in the utmost at the end of the chromosome. A word cloud was generated by taking all the cis-regulatory motifs predicted in the *CcWRKY* promoter region by using the ‘word cloud’ package of R. The word cloud showed the most frequently occurred motifs in promoter sequences such as CAAT, TATA, TATATA and CAAAT (Figure S4). Diverse kinds of cis-regulatory elements were predicted, and from them 15 most common type cis-regulatory elements were scanned (Table S2). These elements contained five hormone responsive elements included TCA-element (CCATCTTTTT, TCAGAAGAGG; involved in salicylic acid response), TGA-elements (AACGAC; a auxin-responsive element), ABRE elements (ACGTG, CACGTG; involved in abscisic acid response), TATC-box-P-box (TATCCCA, CCTTTTG; involved in gibberellin-response) and CGTCA-motif (CGTCA; involved in the MeJA-response); three light-responsive elements G-box (TACGTG, CACGTG), TCT-motif (TCTTAC) and GT1-motif (GGTTAA); a defence and stress receptive element, TC-rich repeats (GTTTTCTTAC); a low temperature responsive element, LTR-WRE3 (CCGAAA, CCACCT); a regulatory element necessary for the anaerobic instruction, ARE (AAACCA); an element concerned with drought-response, MBS (CAACTG); a regulatory element responsive for meristem expression, CAT-box (GCCACT) and a promoter element about -30 base pairs from transcription start site, TATA-box (TATA, TATAA). In the GO enrichment analysis, targeted *CcWRKY* genes were grouped into 15 different functional categories of three major categories; biological processes, molecular function, and cellular components (Figure 5 and Table S3).

### 2.5. Tertiary Structures of CcWRKY Proteins

**Figure 6:**
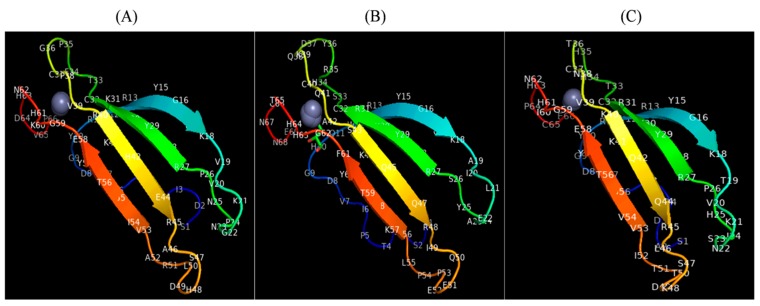
The homology-based tertiary structure of three WRKY proteins representative of three major groups of *WRKY* genes identified in pigeonpea genome. The protein structure of each of the three (**A**) CcWRKY1, (**B**) CcWRKY2 and (**C**) CcWRKY4 were modeled with greater than 90% confidence score. Potential active sites are shown in these structures.

A total of five 3D structures for the C-terminus domains identified in CcWRKY1, CcWRKY2, and CcWRKY4 was predicted using the I-TASSER software. The 3D model having the highest C- score was selected as the most accurate structure, and used for further analysis (Table S4). The confidence (C) score is used to estimate the overall model quality of the I-TASSER model. This C-score is basically based on the consequence of threading template alignments and total coverage of the structure. TM-score and RMSD are known standards for determining structural similarity, is the amount of global structural resemblance between query and templates. The protein structures of each of the three CcWRKY- groups were modeled with greater than 90% confidence score. The stereochemical properties and reliability of the predicted CcWRKY models were validated by subjecting the PDB files to the PROCHECK component of the PDBSum server. For backbone conformation analysis, the predicted models were further inspected by the assessment of ϕ (phi) and ᴪ (psi) backbone dihedral angles as mentioned in the Ramachandran plot. The binding sites of the target protein were predicted by using the COACH server shown in the figure (Figure 6).

### 2.6. Expression of *CcWRKY* Genes During Different Developmental Stages

**Figure 7:**
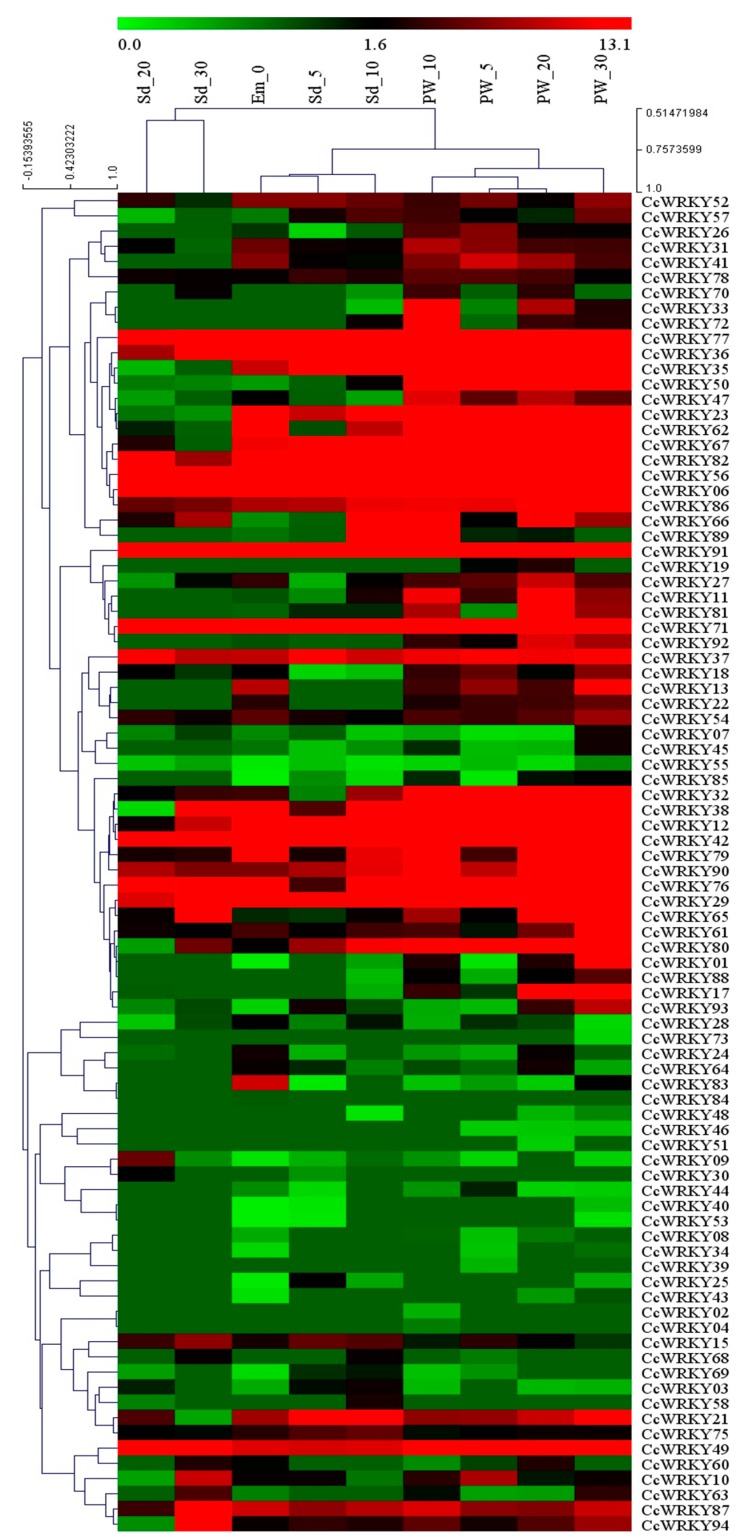
Heat map showing the expression of *CcWRKY* genes at different developmental stages. The heat map was generated by using fragments per kilobase per million mapped reads (FPKM) values obtained from different developmental stages, embryo sac from the day of anthesis (0 DAA), seed (5, 10, 20, 30 DAA) and pod wall (5, 10, 20, 30 DAA) of pigeonpea cultivar “Asha” (ICPL 87119).

The transcript abundance analysis of *CcWRKY* genes during embryo sac, seed and pod wall development in pigeonpea were analyzed using transcriptomic data (BioProject: PRJNA344973 with nine Sequence Reads Archive (SRA) samples). Evidence of the gene expression based on expression in at least one stage was observed for 88 out of 94 *CcWRKY* genes (Figure 7). The *CcWRKY* genes showed a broad range of expression spectrum throughout the plant developmental stages. Some *CcWRKY* genes like *CcWRKY06*, 29, 42, 56, 71, 77 and 91 showed a relatively higher expression level in all developmental stages, while the gene *CcWRKY55* expressed much less. The hierarchical clustering of *CcWRKYs* expressed in the embryo sac, seed and pod wall showed four main clusters on the basis of expression pattern (Figure S5). Most of the genes that showed quite similar expression patterns were clustered together, while the other genes produced a separate cluster.

Similarly, the RNA Seq data (Bioproject: PRJNA354681with 30 biosamples) were used to study the comparative expression profiles of the identified *CcWRKYs* in different tissues (Figure S6 and Table S5). Our analysis showed that tissue-specific expression pattern exhibited by certain *CcWRKY* members. Six of 94 identified *CcWRKYs* shows constitutive expression throughout the different developmental stages while *CcWRKY19* and *CcWRKY20* showed no expression. Additionally, three *WRKY* genes namely *CcWRKY16*, *CcWRKY29*, and *CcWRKY41* were expressed particularly in root nodules (Figure S6 and Table S5), signifying their promising role in symbiosis with the rhizobial bacteria. 

### 2.7. Gene Co-expression Network of CcWRKY Proteins in Pigeonpea

For the co-expression analysis between *CcWRKY* genes, normalized expression data were used. To determine the correlation of the *CcWRKY* genes with other pigeonpea genes, pearson correlation coefficient (Pearson r) was used with default parameters as lower percentile rank 5 and upper percentile rank 95. The entire list of predicted *CcWRKY* genes were used as bait genes and Plaza families were chosen as a reference for the gene family. A total of 93 CcWRKY proteins showed co-relation with each other in pigeonpea genome (Figure S7). Among them, CcWRKY2, CcWRKY12, CcWRKY15, CcWRKY39, CcWRKY80, and CcWRKY85 shows a robust correlation with other CcWRKYs, whereas CcWRKY8, CcWRKY11, CcWRKY13, and CcWRKY87 showed a very low level of correlation among the CcWRKYs. 

### 2.8. Expression of *CcWRKYs* Under Drought and Salt Conditions

**Figure 8:**
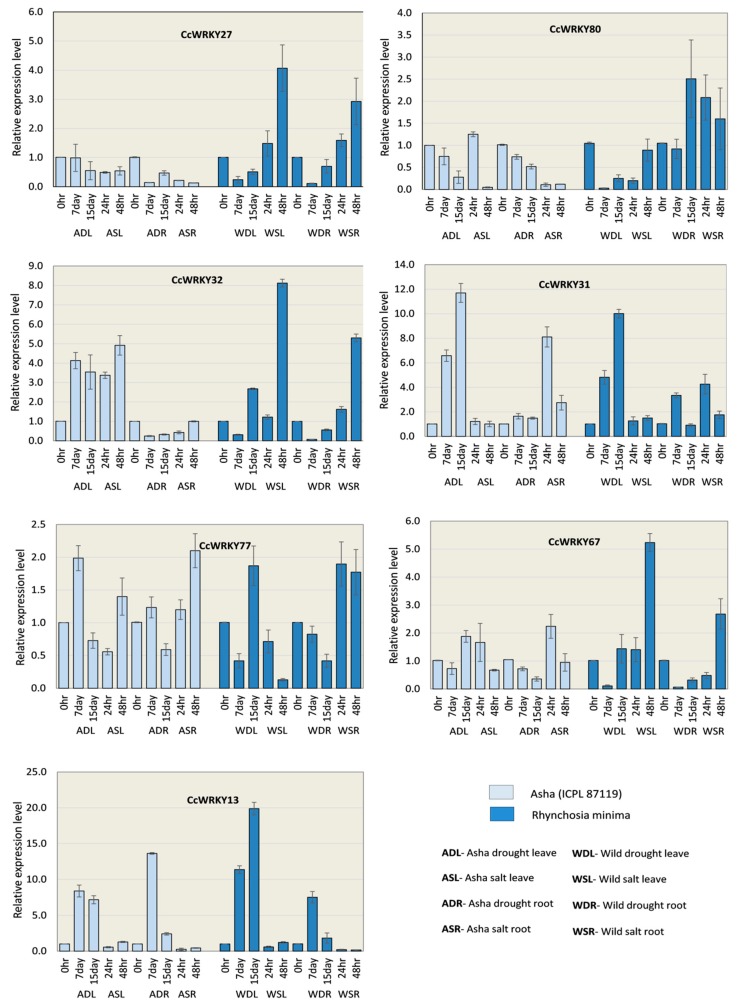
Expression profiling of seven *WRKY* genes in various tissues of pigeonpea grown under drought and high salinity stress. The expression level of these *CcWRKY* genes was investigated in leave and root tissues of two different genotypes Asha (ICPL 87119) and *Rhynchosia minima* (Wild) in response to drought and salinity treatments. All experiments were conducted with three biological replicates.

*WRKY* genes are well-known to have a role in various abiotic and biotic stress tolerance mechanisms. Out of 94, seven *CcWRKYs* were selected for qRT-PCR analysis based on their expression patterns in RNAseq expression analysis. The expression patterns of selected *CcWRKY* genes were studied in two genotypes Asha (ICPL 87119) and *Rhynchosia minima* (Wild) in response to drought and salinity stress treatments. For drought treatment, samples of root and shoot from both the genotypes at 7 days post inoculation (dpi) and 15 dpi were used for this analysis, whereas, 24 h and 48 h salinity treated samples of root and shoot were taken for the analysis. Details of primers used in the qPCR study are provided in Table S6. The WRKY gene *CcWRKY27* was found to be up-regulated in root and leave tissues of wild samples, while in Asha, expression of the gene was recorded as down-regulated in all the samples (Figure 8). Similarly, *CcWRKY80* also showed down-regulation in Asha, however, the gene had mix expression pattern (both up and down-regulation in different stages of same tissue against a treatment) in wild samples. Another WRKY *CcWRKY32* gene found to have mix type expression pattern, up-regulated in leaf and down-regulated in root at all stages in response to all the treatments, except wild salinity root tissue, in which the gene showed up-regulation. The gene *CcWRKY31* was found to be highly up-regulated in most of the tissues, while it showed non-significant expression in a few tissues like Asha drought root (ADR), Asha salinity leaf (ASL) and wild drought root (WDR). Another gene *CcWRKY77* was also up-regulated in Asha against both stresses, while, the gene was found to have mix expression pattern in wild tissues (Figure 8). The mix expression pattern was also observed for *CcWRKY67* in all tissues, besides wild leaf and root tissues treated with salinity, in which the same gene showed up-regulation in leaf and root tissues. The last gene of the order, *CcWRKY13* was shown to be up-regulated in both leaf and root against both stresses of drought and salinity. It also had a mix expression pattern in Asha salinity tissues and down-regulated in wild salinity tissues (Figure 8).

## 3. Discussion

Pigeonpea is an essential food legume crop which provides a protein-rich diet for more than a billion people. Considering its importance in dryland agriculture, efforts have been made to sequence the entire genome of pigeonpea. The availability of the high-quality genome sequence of pigeonpea provides an opportunity to perform the identification of genes, their functional annotation and also to understand the evolution. In this regard, genomic and transcriptome resources available for pigeonpea were efficiently utilized here to study the *WRKY* gene family. Earlier studies have confirmed that the WRKY transcription factor plays a vital role in regulating different physiological processes, such as plant growth, development, plant senescence, signal molecule delivery, biotic or abiotic stress responses, and synthesis of resulting metabolites. The WRKY TFs are localized in the nucleus and bind with the W-box promoter of the targeted gene to regulate the gene expression under stress conditions.

In recent years, a considerable number of WRKY TFs have been identified and analyzed in various plant species through genome-wide studies. The number of *WRKYs* varies from species to species. Structural and functional studies of WRKYs have been conducted in various plants species. However, no genome level study on WRKY identification in the pigeonpea has been reported to date. In our study, we report for the first time a genome level study of *WRKY* genes in pigeonpea and also investigated the functional structure of WRKY gene family in pigeonpea legume crop. The pigeonpea with 94 *WRKY* genes is the third largest *WRKY* gene family containing legume crops after the plant species such as soybean (174 members) and *Medicago truncatula* (98 members) (Figure S8). The *WRKY* gene numbers are not evidently co-related with the genome size. In peanut (*Arachis hypogaea*) only 28 *WRKY* genes were present, which have a genome size of ∼2.7 giga bases (Gb), while soybean has 174 members of *WRKY* genes in 1.1 Gb genome [58,59]. 

Comparative structural analysis of pigeonpea WRKY proteins by sequence alignment and phylogenetic examination showed some interesting insights. A phylogenetic tree of 428 WRKY TFs was generated from four species (*C. Cajan*, *G. max*, *P. vulgaris,* and *A. Thaliana)* consistently showed that all *WRKY* gene were clustered into I, II and III groups, with subgroups of group II named as IIa–IIe. The C-terminus WRKY domain of group I is very close to the subgroup IIc (Figure S3), and group IId and IIe also seemed to have a common origin. In generally it revealed that group IIc WRKYs might evolve from group I WRKYs. Additionally, 82 other types of domain were found in CcWRKYs (Pigeonpea WRKY), signifying their diverse functions, which lead to variations at their structural and physiological levels. We analyzed the conservation of WRKY domain, an important motif of the *WRKY* gene in all the WRKY groups and found specific group-specific domain sequence variants in the legumes (Table S7) [28]. In comparison with our findings, nine and three WRKY domain variants have been reported in rice and *Arabidopsis*, respectively [56,60]. The variation in the WRKYGQK motif may affect the DNA-binding specificity of WRKY proteins. A DNA-binding study revealed that a Q/K substitution of amino acid residue change in the WRKYGQK heptapeptide motif might have altered the DNA-binding abilities of the respective *WRKY* genes [61]. In soybean, *GmWRKY21* and *GmWRKY6* genes contained the WRKYGKK variant, which does not bind to the W-box [15]. Similarly, *GmWRKY167* contained a WRKY domain and an N-terminal Golgi-targeting transmembrane domain which is recognized only in legumes. The conserved WRKYGQK motif of GmWRKY167 was altered into WRKYEDK, results in the recombinant GmWRKY167 domain has no binding specificity for W-box sequences [40]. Tobacco *NtWRKY12* gene contained the WRKYGKK variant, which recognizes different binding sequence TTTTCCAC in place of regular W box [62].

In our study, eight WRKY genes (*CcWRKY09*, *CcWRKY10*, *CcWRKY17*, *CcWRKY59*, *CcWRKY66*, *CcWRKY70*, *CcWRKY72*, and *CcWRKY89*) have a single amino acid mismatched in their conserved WRKYGQK heptapeptide (Figure S2). The remaining two WRKY variants contained a substitution of two amino acid in their WRKYGQK signature motif, while one WRKY gene *CcWRKY11* contains 4 amino acid substitutions that changed WRKYGQK to WKKFEDK, which might be altered or even rejected their DNA-binding specificity as it is discussed earlier in soybean WRKY gene *GmWRKY167* [44]. Pigeonpea WRKY proteins along with altered DNA-binding specificity may lead to the new molecular activities and biological functions. The structural alterations of the pigeonpea *WRKY* genes may lead to their functional diversification and also facilitate to identify the association of similar function activities of *WRKY* in other crops.

The percentage gene dispersal among different subgroups showed the distribution of the *WRKY* genes in related leguminous species, i.e., *G. max*, *C. cajan*, *P. vulgaris* and *A. thaliana* that is found to be highly comparable among the plants (Figure 2B). Mostly, in dicotyledonous legume crops, group IIc holds the higher number of WRKY members than other groups. In the case of *G. max*, *C. cajan*, and *P. vulgaris*, the group IIc is the largest. In our results, 24.4% of the total number of *CcWRKY* genes occurred in group IIc (Figure 2A). We observed a substantial variation in CcWRKY proteins by analyzing the protein isoelectric focusing point, aliphatic index, instability index, and molecular mass. In addition, gene structure analysis of *CcWRKYs* showed that the *CcWRKYs* belongs to a similar group sharing common exon-intron structures. In our study, the numbers of introns in group IIc and IIb members are comparatively higher than other group members (Figure 3). These results indicate that loss or gain of exons and introns occurred in the group I and II *CcWRKY* genes during the evolution, which may lead to the functional diversity of the closely correlated *WRKY* genes [63]. The cis-regulatory elements that occurred in the promoter sequences are principal molecular switches participated in the transcriptional control of a gene by controlling an extensive gene network which is involved in diverse biological processes including developmental processes and stress responses [56]. In the present study, the identification of prominent cis elements in the 2 kb upstream region of pigeonpea *WRKY* genes indicates the connection of the respective genes in various stresses like drought, heat, salinity, light, salicylic acid, abscisic acid, auxin, gibberellin as well as pathogen resistance (Table S2). 

The WRKY gene family extended and diversified through the evolution processes from the green algae to plants. Gene expansion and diversification happened more rapidly in seeded plants than others [64]. The tandem and segmental duplication play a critical role in the extension of *WRKY* genes which is evident from the genome-wide analysis performed in *Arabidopsis, soybean* and *B. distachyon* [65,66,67]. In case of pigeonpea, six tandem duplication events in *CcWRKY* genes that arranged in three different clusters were observed (Table S8). This suggests the unanimous mechanism involving tandem duplication, particularly in legume plant species. 

Several pieces of evidence have demonstrated that WRKY TFs are involved in the stress tolerance mechanism. Approximately 154 *GmWRKY* genes in *Glycine max* [12] and 54 *OsWRKY* genes in *Oryza sativa* are differentially expressed under abiotic stresses [68]. Similarly, 14 *MrWRKY* genes in *Medicago ruthenica* responded to abiotic stress treatments [69]. In soybean, *GmWRKY13*, *GmWRKY27*, and *GmWRKY54* genes play differential roles during abiotic stress tolerance [22]. Moreover, *GmWRKY13* is reported to participate in the abiotic stress response as well as lateral root development. In the same study, *GmWRKY27* is reported to exhibit more tolerant to cold stress, but not to drought or salt, while *GmWRKY54* had greater association with tolerance against drought and salt stresses [22].

The present study and most of the previous studies with WRKY family in other plant species have mostly addressed genomics and transcriptomic aspects. There is much less information about functional regulation of the WRKY gene family at protein level. Therefore, more extensive efforts are needed with proteomics to better understand the regulation of the WRKY gene family in plants. Recent development in proteomics tools provides an opportunity to perform high-throughput studies in plants. Such advances expected to generate a significant amount of protein data which can be integrated with transcriptomics and genomics to gain a global prospective of the complex regulation system of WRKY. 

*WRKY* gene expression profiling has been performed in different crop species like rice (13 *OsWRKY* genes) and wheat (15 *TaWRKY* genes) under the response to different abiotic stresses gives the insight to understand the molecular biological function of the genes [19,20]. For the functional characteristics of the pigeonpea *WRKY* genes, we have analyzed the tissue-specific expression pattern of 94 identified *CcWRKY* genes in five different developmental stages like germinal, seedling, vegetative, reproductive and senescence stages using the RNA sequenced data (Figure S6). Pigeonpea *WRKY* genes belonging to the group I and IId showed exponentially high expression in different plant tissues (Figure 7). In particular, a greater proportion of group I and IId *CcWRKY* genes was expressed at their higher level in the entire plant tissues including embryo, seed and pod wall, whereas the expression level of *CcWRKY* genes in other groups was found to be comparatively very low in all the tissues (Figure 7). The biological significance of the group IId *CcWRKY* gene transcripts is still unclear. Members of this *WRKY* group were showing high expression in all the tissues that might play a role in pigeonpea growth and development. Additionally, some *WRKY* genes were exclusively expressed in the pod wall (Figure 7), which may suggest their possible role in pod and seed development. Our analysis revealed that the tissue-specific expression pattern exhibited by certain *CcWRKY* members. Six of 94 identified *CcWRKYs* showed constitutive expression throughout the different developmental stages, while *CcWRKY19* and *CcWRKY20* showed no expression at all. Using qRT-PCR, we have also analyzed seven pigeonpea *WRKY* genes to evaluate their expression pattern in both leave and root tissues in response to drought and salinity (Figure 8) and found that *CcWRKY32* was up-regulated in wild samples, while it was down-regulated in Asha against salinity stress, suggesting the importance of the gene during salt tolerance in pigeonpea. The wild genotype used in this current study has a great extent of drought and salt tolerance in comparison with cultivated Asha genotype of pigeonpea. Similarly, *CcWRKY80* had a mix expression pattern in the wild, whereas its down-regulation was observed in Asha, which clearly indicates the significance of this gene in the abiotic tolerance. However, *CcWRKY67* and *CcWRKY13* contrastingly expressed in wild type genotypes to sustain the salt toxicity. These findings could be utilized in pigeonpea crop improvement program to develop salt and drought tolerance variety for getting high yields. 

## 4. Materials and Methods

### 4.1. Identification of Genes Encoding WRKY Transcription Factors (TFs) in Pigeonpea Genome

To identify the putative WRKY TFs present in the pigeonpea genome, draft genome sequence data of the pigeonpea genotype ‘Asha’ (ICPL 87119) was used [5]. Initially, 994 full length amino acid sequences encoding WRKY TFs from 10 different plant species (*Arabidopsis thaliana*, *Oryza sativa sp. Japonica*, *Brachypodium distachyon*, *Populus trichocarpa*, *Zea mays*, *Vitis vinifera*, *Glycine max*, *Fragaria vesca*, *Mallus domestica* and *Medicago truncatula*) were retrieved from the Plant TFs Database [70] and used as a query to identify homologs from pigeonpea proteome by using BLASTP (https://blast.ncbi.nlm.nih.gov) search using default parameters. Furthermore, an Hidden Markov Model (HMM profile (PF03106) for the WRKY domain was used to screen for the presence in the entire predicted pigeonpea proteome using the HMMER program (v3.1b2; http//hmmer.janelia.org/). The results were collectively used for the presence of WRKY domains using ScanProsite (http://prosite.expasy.org/scanprosite/) and the NCBI-Conserved Domain Database [71]. The WRKY identified in pigeonpea was designated as CcWRKY. The CcWRKY proteins without a conserved WRKYGQK domain were discarded.

### 4.2. Sequence Analysis and Phylogeny

The WRKY family members of *Glycine max* were retrieved from the Plant Transcription Factor Database [70]. The multiple sequence alignments of *G. max*, *M. truncatula*, and pigeonpea were performed using Clustal X v2.1 using default parameters [72]. Sequence alignment was used to generate a phylogenetic tree using the MEGA6.06 software tool. The neighbor-joining method with 1000 bootstrap replicates was used to confirm the robustness of each node in the phylogenetic tree [73]. The final tree is represented interactively using iTOL (https://itol.embl.de/). All the WRKY TFs were classified into different subgroups based on the alignments and phylogenetic positioning of WRKY protein sequences in pigeonpea and *Glycine max*.

### 4.3. Gene Structure, Motif Conservation, and Regulatory Element Analysis

The composition of deduced CcWRKY proteins with physical and chemical characteristics was analyzed with the Expasy Prot-Param tool (http://web.expasy.org/protparam/). Exon-intron organization of *CcWRKY* genes was performed by comparing CDS sequences with their respective genomic sequences by the Gene Structure Display Server [74]. Conserved motif sequences across the 94 CcWRKY peptides were analyzed by MEME Suite v.5.0.2 [75]. The search criteria taken for the identification of motifs by the MEME includes a maximum of 20 motifs to report, and optimal motif width between 8 to 50 residues. Then the conserved motifs were annotated using InterProScan Search (http://www.ebi.ac.uk/Tools/pfa/iprscan/). The subcellular locations were predicted using WoLF PSORT [76] as well as PlantmPLoc server [77]. The upstream 2 Kb regions of all *CcWRKY* genes were fetched by a custom Perl script and analyzed to determine the presence of *cis*-regulatory elements by the Plant Cis-acting Regulatory DNA Elements (PLACE) database [78]. Then predicted *cis*-regulatory motifs were used to draw a ‘wordcloud’ using a wordcloud package of R.

### 4.4. Gene Annotation and Co-expression Network Analysis

The functional annotation of CcWRKY proteins was performed by Blast2GO Tool [79]. The WRKY protein sequences were imported to Blast2GO program, and executed in three steps: (1) perform BLASTP against NCBI protein non-redundant (nr) database, (2) map Gene Ontology (GO) terms with the BLASTP results, and (3) annotation of GO terms with already known protein functions. The output of this program is categorized into (i) molecular function (ii) biological processes and (iii) cellular components. Comparative Co-expression Network Construction and Visualization (CoExpNetViz) software [80] was used for gene co-expression network analysis and network visualized by Cytoscape v.3.6.1 [81].

### 4.5. Homology-Based CcWRKYs Protein Structure Prediction

Three dimensional (3D) structures modeling of the identified CcWRKY domain sequences was done by I-Tasser (http://zhanglab.ccmb.med.umich.edu/I-TASSER/) with default parameters. Selection of the best model was made on the basis of the highest confidence score. Validation of the predicted 3D model was carried out by PROCHECK unit of the PDBsum server [82] and further re-confirmed by RAMPAGE [83]. Active site prediction was done by COACH-Server (http://zhanglab.ccmb.med.umich.edu/COACH/) and decorated using chimera 1.13.1 (http://www.rbvi.ucsf.edu/chimera).

### 4.6. Transcript Abundance Analysis

Transcript abundance analysis was performed using transcriptome data publically available with the Bio-Project PRJNA344973 and PRJNA354681, containing the data for several tissues of pigeonpea harvested at different developmental stages. Bowtie2 (http://bowtie-bio.sourceforge.net/index.shtml) was used to align SRA reads with pigeonpea draft genome using default parameters. The transcript abundance of *CcWRKY* genes during different developmental stages was estimated by calculating the read density of mapped reads as ‘fragments per kilobase per million mapped reads’ (FPKM) [84]. The heatmap was generated using Multi-experiment Viewer (http://www.tm4.org/).

### 4.7. Plant Materials and Stress Treatments

For the gene expression study, Asha (ICPL 87119), a mild period, high yield variety, and a wild variety *Rhynchosia minima* were selected. The pure seeds for Asha and wild variety were collected from the National Research Centre on Plant Biotechnology and the division of genetics, Indian Agricultural Research Institute (IARI), Pusa New Delhi. Mature seeds were thoroughly washed by diethyl pyrocarbonate (DEPC) water, then soaked overnight. The germinated seedlings were planted in 4-inch plastic pots which contain a mixture of autoclaved black soil, sand, and vermicompost (10:10:1 *v/v*) [85]. The plants were grown under controlled greenhouse conditions with three replications.

Drought treatment was imposed on 15 day-old plants. The drought severity was maintained by adding the exact amount of water transpired from plants and evaporated from the soil surface by weighing the pots regularly. The control plants were maintained at about 80% of relative water content (RWC), while stressed plants were dried up to 30% RWC [85]. Leaf and root samples were collected at 0 day, 7th day and 15th day after the drought treatment started. RWC was calculated by the method of previously described by Schonfeld [86]. 

For salt treatment, 15 day-old plants were moved into a hydroponic system supplemented with 150 mM NaCl solution (Electrical Conductivity (EC) value 16.2), and tissues were harvested at 0 h, 24 h, and 48 h intervals after the stress treatment. Fresh leaves and root tissue samples were harvested from each treated and control plants and immediately frozen in liquid nitrogen. All the samples were stored at −80 °C until RNA isolation.

### 4.8. RNA Isolation, cDNA Synthesis, and qRT-PCR

Total RNA was isolated from all drought and salt-treated samples by using Spectrum Plant Total RNA Isolation Kit (Sigma-Aldrich, St. Louis, MO, USA) and single-stranded cDNA synthesis was done using iScript cDNA Synthesis Kit from Bio-Rad, following the manufacturer’s guidelines. Gene-specific primer pairs were designed by Primer3 online software (http://bioinfo.ut.ee/primer3-0.4.0/), and further verified their specificity by observing a single distinct band in gel-electrophoresis. The qRT-PCR run was performed by using Bio-Rad SYBR® Green master mixes on an Applied Biosystems™ 7500 Fast Real-Time PCR System (Waltham, MA, USA), as per the ABI protocol. The amplification parameters were 95 °C for 2 min, 40 cycles at 94 °C for 15 s then 60 °C for 30 s, and melting curve temperature ramping from 65 °C to 95 °C with fluorescence detection at every increment of 0.5 °C. The comparative gene expression analysis was done by the 2-∆∆CT method [87]. 

## 5. Conclusions

In the present study, extensive analysis of the WRKY TF gene family in pigeonpea identified 94 *WRKY* genes which are comparable to the number of *WRKY* genes identified in the other species belonging to the Fabaceae family. The chromosomal localization, phylogenetic distribution, exon-intron structure analysis, and prospective motif composition provided a basis for the understanding of *WRKY* gene family evolution. The analysis of cis-regulatory elements in 2 Kb upstream regulatory regions revealed the occurrence of some important motifs related to WRKY functions under biotic and abiotic stress responses. Gene ontology analysis revealed gene distribution at a greater range of biological processes, molecular functions as well as cellular components. The expression analysis of *WRKY* genes in two different genotypes at leaf and root tissue in response to drought and salt stress were studied. The computational study showed that *CcWRKYs* of the same group reveal similar physicochemical properties. The qRT-PCR analysis results suggested the possible role of differentially expressed *CcWRKY* genes under different abiotic stresses. This study generated an important resource that will provide helpful information for further exploration of the *CcWRKY* TFs role in the regulatory mechanism in response to abiotic stresses. The present study can also provide a reference for future structural, functional investigations of WRKY TFs and molecular breeding of pigeonpea plant.

## Supplementary Materials

The following are available online at https://www.mdpi.com/2223-7747/8/7/214/s1: Figure S1: Confirmation of the presence of conserved WRKY domain by CDD search. All the 97 predicted *WRKY* genes searched for the presence of conserved WRKY domain using CD-Search Tool (https://www.ncbi.nlm.nih.gov/Structure/bwrpsb/bwrpsb.cgi); Figure S2: Multiple sequence alignment of pigeonpea WRKY domain sequences. The sequence alignment of identified 94 *WRKY* genes was performed using clustalX. suffix “C” or “N” indicated the C-terminal and N-terminal domain sequences of the WRKY proteins. At the below blocks shows the conservation of amino acid motifs across all sequences; Figure S3: Unrooted phylogenetic tree were constructed using *WRKY* genes from the complete WRKY gene of four crops. Full length amino acid sequences of WRKY of pigeonpea, *Glycine max*, *Phaseolus vulgaris* and *Arabidopsis thaliana* were aligned with Clustal X and the phylogenetic tree was generated by the neighbor-joining method with 1000 bootstrap replicates using the MEGA 7.0 software tool; Figure S4. Word-cloud showing the cis-regulatory elements identified in the 2kb upstream promoter regions of the 94 *WRKY* genes in pigeonpea genome. The intensity and size of the motifs indicate their frequency of occurrence; Figure S5: Hierarchical clustering of *CcWRKY* genes. Clustering of *CcWRKY* genes expressed in embyo sac, seed and pod wall. RNA-seq data obtained from the Sequence Reads Archive (SRA) database with Bio Project accession PRJNA344973 and cluster analysis was performed using MeV (http://www.tm4.org/mev.html); Figure S6: Expression of *CcWRKY* genes at germinal, seedling, vegetative, reproductive and senescence stages. Heat map showing the expression pattern of *CcWRKY* genes at different developmental stages like, germinal (tissues: cotyledons, radical, hypocotyl and embryo), seedling (tissues: shoot and root), vegetative (tissues: SAM, nodule, root and leaf), reproductive (tissues: leaf, bud, flower, stamen, pistil, root, nodule, immature pod, stem, petiole, petal, sepal, SAM, mature pod, immature seed and mature seed) and senescence stage (tissues: petiole, leaf, root and stem) of pigeonpea “Asha” (ICPL 87119) variety; Figure S7: Gene Co-expression network of *CcWRKY* genes. Co-expression network of pigeonpea *WRKY* genes constructed using the publically available RNA-seq data (Bio-project accession PRJNA344973 and PRJNA354681). The network was generated with Comparative Co-Expression Network Construction and Visualization tool (CoExpNetViz) and visualized by Cytoscape V.3.6.1. Green and red lines denote positive correlation and negative correlation, respectively; Figure S8: Genome-wide distribution of *WRKY* genes in *Fabaceae* species and *A. thaliana*. Phylogeny is based on NCBI taxonomy and constructed by using phylogenetic tree generator; Table S1: Physico-chemical properties of CcWRKY proteins. Detailed information of 94 *CcWRKY* genes including conserved domains, domain distribution pattern, phylogenetic grouping, polypeptide physiochemical properties and homology with *Arabidopsis* WRKY proteins for the 94 *WRKY* gene identified in pigeonpea genome; Table S2: Distribution of cis element in 2 Kb upstream regulatory region. The distribution of top fifteen important regulatory motifs in 94 WRKY proteins of pigeonpea; Table S3: BLAST2GO annotation of CcWRKY proteins. Categorization of annotated pigonpea WRKY proteins in three different categories, biological processes, molecular function and cellular components, Table S4: Protein model quality scores. The quality scores of WRKY protein three dimensional models predicted by using the homology-based approach, Table S5: FPKM values of the WRKY genes predicted from the publically available RNA-seq dataset of different tissue samples collected at different developmental stages. Table S6: Primer list. List of primer sequences used for real-time PCR analysis of selected *WRKY* genes of pigeonpea, Table S7: Group specific WRKY variants predicted in legume crops, Chickpea (*Cicer arietinum*), Soybean (*Glycine max*), Pigeonpea (*Cajanus cajan*), and Barrel medic (*Medicago truncatula*), Table S8: List of randomly duplicated genes in pigeonpea predicted from the Plant Tandem duplicated Gene Database PTGBase http://ocri-genomics.org/PTGBase/.
